# Supplementary material for: Are carbon emissions trading and green financial instruments synergistic? -Comprehensive quantitative research based on content analysis
Source: PLoS One. 2024 Mar 7;19(3):e0298601. doi: 10.1371/journal.pone.0298601 (PMC10919601; doi:10.1371/journal.pone.0298601)
Supplement: S1 Data — (ZIP) [file pone.0298601.s001.zip › Data/地级市Z_F工作报告.zip!/╔┬╬≈/═¡┤¿╩╨/└┤╫╘╣1⁄2╓┌║┼í╛┬φ┐╦╩2╛▌═°í┐_2007.docx]

各位代表：

　　现在，我代表市人民政府向大会作工作报告，请予审议，并请市政协委员和其他列席人员提出意见。

　　一、2006年工作回顾

　　过去的一年，在市委的正确领导下，全市人民坚持以邓小平理论和“三个代表”重要思想为指导，牢固树立和落实科学发展观，坚持“5523”工程发展思路，团结拼搏，克难攻坚，全面完成了市十四届人大一次会议确定的各项目标任务，实现了“十一五”经济社会发展的良好开局、高点起步！

　　（一）国民经济持续快速增长。实现生产总值83．63亿元，增长15％，增幅居全省第三位。财政总收入 8．49亿元，增长34％，其中地方财政收入4．07亿元，增长34．5％，增幅居全省第三位。规模以上工业总产值96．89亿元，增长36．7％，增幅居全省第三位。主要工业品产量原煤1677万吨、水泥566万吨、电解铝13万吨，分别增长22.4％、14．7％和81．1％，工业综合效益指数122，较上年提高31．5个百分点。社会消费品零售总额25．4亿元，增长13．8％。

　　（二）重点项目建设成效显著。固定资产投资完成43．73亿元，增长26．8％，增幅居全省第四位。“十一五” 第一批21个重大项目完成投资18．4亿元。铜川铝业节能技改、照金煤矿改扩建、声威100万吨、建秦50万吨水泥粉磨站、东塬 330KV输变电工程、柳照路等项目建成运营。华能铜川电厂完成投资10亿元，主厂房已封顶；玉华煤矿改扩建、中星新材料研发基地一期工程等项目进展顺利。铜川铝业节能技改续建、西川煤矿、秦岭20O万吨水泥粉磨站、30万吨水煤浆生产线、市一中新区校区等项目开工建设。

　　（三）新农村建设扎实起步。编制了新农村建设规划，启动了51个新农村示范村建设。全市用于“三农”的支出2．99亿元，是历史上最多的一年。粮食总产24．83万吨，较上年增长4．8％；创历史最好水平。新栽干杂果11．5万亩、水果4．8万亩，累计达到84．3 万亩和49.1万亩，农民人均达到3亩果。苹果总产29．85万吨，增长17％，优果率68％，铜川苹果荣获2008北京奥运推荐果品一等奖和中华名果称号。新增奶牛3008头，奶牛存栏l．34万头；牛奶产量1．78万吨，增长 53％，一批奶牛、苹果等专业村脱颖而出。建成通村油路和水泥路467．8公里，一年修了十年的通村路。又有3．8万农村人口解决了安全饮水问题，新建沼气池7050口；移民搬迁6200人，在全省率先实现了户户通电的目标。植树造林21万亩，治理水土流失154平方公里。

　　（四）节能减排力度加大。落实节能降耗责任，实行严格的环保制度，强化指标约束，单位生产总值能耗有所下降。关闭淘汰3万吨以下小煤矿29个、小造纸厂4家，关闭拆除了3家水泥企业的3条机立窑和2条中空窑生产线。整顿规范矿产资源开发秩序取得阶段性成果。改造燃煤锅炉30台，清理青岗岭、黄堡、柳湾等地的煤场76家，煤烟污染治理迈出新步伐。全市化学需氧量削减2％，市区空气质量二级和好于二级的天数达到245天。

　　（五）创卫工作开局良好。制定了创卫规划和实施方案，加大宣传力度，强化城市管理，创卫的良好氛围已经形成。投入1．5亿元加强了城市道路、排水、公厕、垃圾和污水处理等基础设施建设，投入570万元改善了环卫装备，新耀垃圾填埋场投入试运行，新耀污水处理管网工程开工建设。编制了城市分区规划和绿化等专业规划。新区长虹路Ⅱ标段、咸丰路至景丰中学道路改造、华原东道照亮等工程竣工，南部工业园区排水工程开工建设。沮河橡胶坝建成蓄水。百盛国贸大厦、正大商城建成运营，漆水河景观改造完成工程量的83%，新建城市街头广场5处。210国道环境整治取得阶段性成效，川口地区的环境状况有了显著改善。我市被省政府授予城市环境综合整治先进市称号。

　　（六）改革开放步伐加快。15户国有企业近2亿元的债务通过打捆回购等手段得到化解。完成了14户国有企业的改制任务，东风机械厂政策性破产程序终结。国有粮食购销企业改革基本完成。新登记个体工商户1850户、私营企业380户。政府预算收支分类改革顺利完成，市本级部门预算扩大到18个试点单位，在10个部门进行了国库集中支付改革试点。消化政府到期债务2421万元。全年招商引资签订合同项目 127个，实际到位资金8.1亿元；有88个项目开工建设，47个项目建成投产。外贸进出口2934万美元，增长21%。接待国内外游客320万人次，增长10%；实现旅游综合收入 3．46亿元，增长15%。

　　（七）社会事业加快发展。全年转化科技成果10项，宜君县被列为国家科技富民强县试点。农村义务教育“两免一补”政策得到全面落实，为9．87万名学生免除学杂费2005万元，为2．99万名贫困中小学生免费提供了教科书，为 1．94万名农村贫困寄宿生补助生活费697万元。投入112万元配备了取暖设施，初步解决了农村小学生的取暖问题。为4000名城市低保家庭学生免除杂费80万元。25所省属企业学校顺利移交地方管理。铜川职业技术学院在校学生达到1700人。各级财政用于新型农村合作医疗的支出1256万元，全市新型农村合作医疗参保人数27．9万人，参合率 90．4%，3．99万人次享受医疗补助1094万元。市医院新区分院主体工程完工，王益区、耀州区人民医院改造工程完工。成功举办了铜川市首届文化艺术节，耀州窑陶瓷烧制技艺被列入全国第一批非物质文化遗产保护名录。新增有线电视用户8500户、数字电视用户2000户。新区体育馆主体工程完工，在省第十三届运动会上取得了 14枚金牌、19枚银牌、24枚铜牌的好成绩。稳定低生育水平工作继续推进，人口自然增长率4．42‰。

　　（八）人民生活水平普遍提高。各级财政用于再就业的资金达到2350万元，增长3倍，是历年来投入最多的一年。全年新增城镇就业1．31万人，劳务输出4．84万人，超额完成了任务。为2.65万名企业离退休人员发放养老金2．2亿元。工伤保险、生育保险参保人数新增9500人和5900人。全市开工住房25．4万平方米，建成19．8万平方米，其中廉价廉租房13．8万平方米，安置住房困难群众1634户。城镇居民人均可支配收入6820元，增加近千元，农民人均纯收入2215元，增加超过200元，增幅居全省第一位、第二位。

　　（九）构建和谐社会稳步推进。市委、市政府为全市人民承办的20件实事圆满完成。城市低保标准由每人每月135元提高到160元，为7．86万名城市低保对象发放低保金6008万元，做到了应保尽保。市财政投入521万元，给城市低保户发放了取暖补贴。农村居民最低生活保障制度启动实施，1．45万人从中受益。以“八荣八耻”为主题的社会主义荣辱观教育活动深入人心，市民的文明素质有所提高。双拥共建活动蓬勃开展，我市荣获省级双拥模范城光荣称号。认真落实市人大及其常委会决议，自觉接受社会各界监督，广泛听取人民群众对政府工作的意见和建议，全年共办理人大代表建议48件、政协委员提案160件。普法工作不断深入，基层民主管理和村民自治得到加强。信访工作力度加大，一批突出问题得到妥善解决。平安铜川创建活动深入开展，社会治安综合治理进一步加强，公共突发事件应急管理工作机制初步建立。加强煤矿安全生产监管的‘十条措施”得到进一步落实，全市安全生产形势总体稳定，安全事故起数和死亡人数分别下降12.6％和17．2%。

　　审计、统计、物价、人防、供销、气象、地震、档案、地方志、外事、侨务等方面的工作都取得了新的成绩！

　　各位代表，2006年是我市经济社会发展最好最快的一年，也是人民群众得到实惠最多的一年！成绩来之不易。这是省委、省政府和市委正确领导的结果，是市人大、市政协和各民主党派监督与支持的结果，更是全市广大干部群众同心同德、拼搏奉献的结果。我代表市人民政府，向奋战在全市各条战线上的工人、农民、知识分子、干部、驻铜部队指战员、武警官兵和公安干警致以崇高的敬意！向大力支持政府工作的各位代表、各位委员和社会各界人士表示衷心的感谢！

　　我们也清醒地认识到，全市经济社会发展中还存在不少困难和问题。一是拉动经济快速增长的动力不足。支撑经济发展的大项目、好项目还比较少，投资快速增长的基础还不牢固。外贸出口总量小，缺乏拳头产品。消费需求不旺。二是经济增长方式依然粗放，原煤、原材料等初级产品多，科技含量和附加值较低，缺乏名牌产品和拥有自主知识产权的产品。三是城市建设欠账多，服务功能不完善；与创建省级卫生城市的目标还有较大差距。四是就业矛盾突出，财政支出压力大，居民收入偏低，食品安全、医疗服务、环境保护等一些涉及群众利益的突出问题，还没有从根本上得到解决，维护稳定的压力较大。五是政府职能转变滞后，监管不到位。一些地方和部门还存在着官僚主义和形式主义，少数干部奢侈浪费，甚至滥用权力。对此。我们必须以对党和人民高度负责的精神，采取更加得力的措施，切实加以解决，决不辜负人民的期望和重托！

　　二、2007年经济社会发展的主要目标任务

　　今年上半年省第十一次党代会将要召开，下半年我们将迎来党的十七大的胜利召开，明年是我市建市五十周年，做好今年的各项工作意义非常重大。铜川正处在难得的快速发展时期。从自身优势看，我市资源丰富，煤炭保有储量29亿吨，石灰石、陶瓷粘土、水泥配料黄土储量居全省前列；城市化水平高，城乡人口基本相当；区位独特，交通便利，是西安大都市圈的重要组成部分；工业化起步早，工业基础较好，具有发展工业得天独厚的条件；铜川是一座新兴城市，外来人口多，具有极大的包容性；城市规划科学，发展空间大，有很大的发展潜力，经国家建设部批准的规划面积55平方公里的铜川新区是各界人士投资置业的理想场所。从外部环境看，经济全球化深入发展，生产要素跨国流动和产业转移步伐加快；我国经济在宏观调控中继续保持平稳较快增长，国家对西部地区的支持力度持续加大；陕西是国内发展较快的省份之一，固定资产投资将保持高速增长，铜川建材工业的优势更加凸现。从内部发展看，近年来重大项目建设积蓄的势能正在逐步释放，经济发展处在上升阶段，生产总值连续三年实现高位增长，呈现出高增长时段拉长的特点。今年又有一批重大项目建成投运，华能铜川电厂两台机组力争实现双投，电力将迅速成长为新的支柱产业；铝业产能将达到25万吨，再经过几年努力，产值有望达到百亿元；秦岭集团水泥生产能力将达到5000万吨，声威第二条日产5000吨水泥熟料生产线上半年开工建设；总投资8．4亿元的铜川矿区采煤沉陷区治理项目全面实施。全市发展的思路更加清晰，重点更加突出，广大干部群众加快发展的愿望更加强烈。我们已经具备了率先发展跨越发展的基础和条件。与此同时，在国家宏观调控刚性约束增强的情况下，土地、信贷闸门趋紧，项目准入门槛提高，经济竞争更加激烈，影响我市改革发展的不确定因素增多。总体来看，机遇与挑战并存，机遇大于挑战，有利因素多于不利因素。只要我们抓住机遇，矢志艰苦奋斗，勇于开拓创新，铜川的未来一定会更加美好！

　　今年全市经济社会发展的总体要求是：高举邓小平理论和“三个代表”重要思想伟大旗帜，以科学发展观统领经济社会发展全局，全面贯彻党的十六大以来中央一系列决策部署，认真落实省委十届九次全会、市第十次党代会和市委十届二次全会精神，紧紧围绕建设以现代建材业为主导的新型工业城市，实现铜川率先发展跨越发展，构建社会主义和谐铜川这一目标，坚持团结稳定和加快发展两大主题，认真贯彻落实宏观调控的各项政策措施，坚持“5523”工程发展思路，继续实施项目带动、工业强市和可持续发展战略，着力打造煤、电、铝、水泥、陶瓷联产联营循环经济产业链，加快经济结构调整和经济增长方式转变，积极推进社会主义新农村建设，认真解决人民群众最关心最直接最现实的利益问题，转变政府职能，改进公共服务，发展社会事业，加强社会管理，维护社会稳定，促进社会和谐，努力实现经济社会又好又快发展，以优异的成绩迎接党的十七大和省第十一次党代会的胜利召开！

　　国民经济和社会发展主要预期目标是：生产总值增长14．7%，达到102亿元；全社会固定资产投资增长20%，达到52亿元；财政总收入增长25%，达到10．6亿元，地方财政收入增长25%，达到5．09亿元；单位生产总值能耗降低4．7%；城镇居民人均可支配收入增长13%，达到7685元，农民人均纯收入增长11%， 达到2460元；城镇登记失业率控制在4．5％以内，居民消费价格总水平涨幅控制在3％以内，人口自然增长率控制在5．5‰以内。

　　做好今年的工作，必须着力处理好以下六个关系。

　　处理好速度与质量、效益的关系，坚持好中求快。加快发展是解决铜川所有问题的关键。我市是速度效益型经济，没有较高的速度就不可能有较好的效益。既要充分利用各种有利条件，努力保持经济较快增长，又要重视发展的质量和增长的效益，注重结构的调整和产业的升级，实现好与快的统一。

　　处理好发展与资源、环境的关系，坚持持续发展。要珍惜资源，保护环境，集约用地，严把项目准入关，做到浪费资源的项目不上，污染环境的项目不上，技术落后的项目不上，不以浪费资源和牺牲环境为代价换取一时的经济发展。

　　处理好新农村建设与城市化、工业化的关系，坚持协调发展。认真落实工业反哺农业、城市支持农村和多予少取放活的方针，扎实推进新农村建设，推动传统农业向现代农业、传统村落向现代村镇、传统农民向新型农民的转变。

　　处理好扩大投资与激活消费的关系，坚持并驾齐驱。坚定不移地实施项目带动战略，着力抓好一批基础设施、重点产业和公共服务项目，夯实率先发展跨越发展的基础。深入挖掘消费潜力，大力培育消费热点，努力扩大消费需求，实现投资消费双拉动。

　　处理好深化改革与扩大开放的关系，坚持统筹推进。向改革要动力，向改革要活力，为落实科学发展观提供体制机制保障。树立开放促进发展的理念，把招商引资作为全局性的战略举措，招商、亲商、安商、富商，努力把铜川打造成招商引资的洼地。

　　处理好经济发展与社会进步的关系，坚持和谐发展。认真解决关系群众切身利益的突出问题，发展社会事业，重视安全生产，化解社会矛盾，努力构建和谐社会。

　　三、切实转变经济增长方式，促进经济又好又快发展

　　（一）以项目建设为主线，增强经济发展后劲

　　我们一定要把项目建设作为总抓手，牢固树立“抓项目就是抓发展、有大项目就有大发展”的理念，集中精力抓项目，为全市经济社会发展奠定坚实的基础！

　　突出抓好重大产业项目。按照“三位一体” 的项目包联责任制，全力以赴加快 10个投资上亿元的重大产业项目建设。力争玉华煤矿改扩建上半年建成试产，秦岭200万吨水泥粉磨站三季度建成投产，铜川铝业节能技改续建、西川煤矿、中星耐磨复合管、旺仔小馒头、30万吨水煤浆、4万吨陶粒砂等项目年内建成投产。开工建设骊山50万吨水泥粉磨站、10万吨碳素等项目，力争开工建设旺仔牛奶、华辰煤层气发电三期、鑫科惠3．6万吨优质铝型材等项目。

　　加快基础设施项目建设。公路建设完成投资2．8亿元，开工建设西铜高速公路改扩建工程，建设药王山旅游公路。加快实施铜川矿区采煤沉陷区治理工程，完成投资4亿元。组织实施好日元贷款、世行贷款、英国政府赠款集中供水项目，建成新区供水二期工程和耀州、印台城市供水工程，自来水户外计量改造2000户。建设新耀天然气二期工程，新增天然气用户3000户。建成稠桑110KV、西南11OKV输变电工程，改造寺沟110KV变电站，完成供电一户一表改造1万户。

　　加强项目前期工作。市财政今年安排400万元作为重大项目的前期费用。要以现代建材业、现代农业、现代服务业和基础设施建设为重点，精心策划、论证、包装、推介一批事关长远发展的重大项目。对华能铜川电厂二期、铜川铝业25万吨电解铝、油母页岩综合开发、陶瓷产业振兴、铝业深加工等重大项目，成立专门的项目推进小组，由市级领导挂帅，配备专门人员，完善激励约束机制，加快项目立项申报等前期准备工作。公布实施全市“十一五”第二批重大项目。

　　（二）以现代建材业为主导，提高工业经济的综合竞争力

　　工业是我市经济发展的主力军。要坚定不移地实施工业强市战略，走新型工业化道路，改造提升传统产业，培育发展新兴产业，不断壮大工业经济的实力。

　　大力发展现代建材业。市财政安排500万元设立现代建材业发展基金，全力推进现代建材业发展。全面实施现代建材业发展规划，加快水泥及水泥制品、铝及铝深加工、高科技材料、建筑陶瓷及陶瓷材料、新型建材业的发展。搞好建材工业园区基础设施建设，提高园区产业承载能力，引导企业集群发展。制定振兴陶瓷业的决定，加强与科研院所的合作，采取政府注资引导、吸引外商投资等办法，扶持培育2一3户有发展潜力的陶瓷企业。坚持质量兴企、名牌强市战略，推进企业技术创新，力争中星新材料研发进入全省“13115”科技创新工程计划，努力把秦岭、声威打造成中国驰名商标。

　　加强工业运行协调。坚持工业运行月通报、季分析制度，突出抓好重点行业、骨干企业和主要产品的跟踪分析，协调解决企业在煤、电、运、流动资金等方面存在的困难和问题。确保全市规模以上工业实现产值125亿元，增长29%。

　　加大国企改革力度。市财政安排500万元作为国企改革费用。成立国企改革攻坚督查小组，加强分类指导，推动未改制企业抓紧改制，督促已改制企业规范运营。启动新川水泥厂、西北耐火材料厂政策性破产，加快秦岭水泥的改革步伐。强化国有资产监管，规范国有企业改制和国有产权转让行为，防止国有资产流失。加强与金融机构的协调，最大限度地核销企业不良债务。

　　各位代表，铜川是一座工业城市。率先发展，首先是工业增长速度的率先，是工业化的率先；跨越发展，核心是产业升级的跨越，是增长方式的跨越。我们一定要从铜川的实际出发，发挥优势，突出重点，以现代建材业为主导，千方百计作好工业经济这篇大文章！

　　（三）以发展现代农业为重点，扎实推进新农村建设

　　“三农”问题是我们全部工作的重中之重。我们要认真贯彻落实中央一号文件，以新农村建设总揽农村工作全局，把发展现代农业作为重要任务，带着对农民的深厚感情，把农业的基础打牢，把农村的事情办好，帮助农民把生产生活安排好，努力促进城乡共同繁荣。

　　加快发展“一村一品”。要尊重农民意愿，搞好优势农产品区域布局，启动实施“一村一品”百村示范、整体推进工程，加快100个特色专业村建设。新栽干杂果 10万亩、水果4万亩，创建10个省市级苹果示范围，果实套袋15亿只，苹果总产31万吨，优果率达到70％。新建标准化奶牛养殖小区10个，新增奶牛3000头。积极发展中药材、蔬菜和烟叶种植，提高种植效益，努力增加农民收入。

　　改善农民生产生活条件。继续搞好51个新农村示范村建设和71个贫困村的扶贫开发，实施20个扶贫开发重点村建设，移民搬迁4900人。全面落实水库移民后期扶持政策。完成宜君县五里镇至黄陵界、王益区青年路至王石凹、梁家塬至陈炉、耀州区孙塬至富平界等通乡油路80公里，新铺通村水泥路200公里，完成通达工程100公里。加快城乡客运一体化进程，建成4个乡镇汽车站。解决20个行政村3万人的饮水安全问题，在40个行政村建设沼气池5000口。新建5个信息化示范乡镇、100个信息化示范村，全市行政村宽带上网覆盖面达到60％。建设基本农田1．3万亩，发展节水灌溉2万亩，改造中低产田 2．3万亩，全市粮食面积稳定在105万亩，总产达到 20万吨以上。

　　深化农村改革。稳步推进乡镇机构、农村义务教育、县乡财政管理体制改革，全面实施乡财乡用县监管制度。清理核实乡村债务，锁定旧债，制止发生新债，积极探索化解乡村债务的措施和办法。推进征地制度、集体林权制度、水权制度改革，完成畜牧兽医管理体制改革。

扶持壮大县域经济。坚持因地制宜、重点突破、整体推进的思路，大力发展县域经济。加快县域工业化进程，积极培育资源型、劳动密集型、农产品加工型产业。完善中小企业信用担保体系，改善企业融资环境，大力发展中小企业和乡镇企业。加快小城镇建设，统筹规划工业园区、集贸市场，促进农村劳动力向非农产业转移、向城镇聚集。扶持农村专业合作经济组织的发展，提高农民的组织化程度，全年培训农民600O人。

　　（四）扩大招商引资，提高对外开放水平

　　适应我国加入世贸组织过渡期结束的新形势，充分利用国际国内两个市场、两种资源，努力实现外向型经济发展的新突破。

　　加大招商引资力度。进一步完善企业为主、政府推动、中介机构共同参与的招商引资机制，突出企业和园区招商引资的主体地位，实施专业招商、产业链招商，着力引进辐射能力强、就业空间大的重大产业项目。组织好西洽会、杨凌农高会等招商活动，有针对性地开展以现代建材业为重点的专题招商，力争在引进知名企业、高科技企业方面实现突破。对规模较大的招商引资项目，坚持一个项目、一套班子、一项制度，加强跟踪协调和服务，提高项目履约率和资金到位率。

　　着力改善投资环境。把投资环境作为生命线工程来抓，强化社会信用，规范行政行为，提高办事效率，营造良好的政策环境、法制环境、人文环境和工作生活环境。将改善投资环境列入年度经济社会发展考核指标体系，夯实主要领导投资环境建设的责任。健全投诉网络及投诉案件查处快速反应机制，严肃查处破坏投资环境的行为，提高投资环境的竟争力和吸引力。

　　进一步扩大对外开放。充分发挥驻外机构的作用，加强同徐州、烟台、泉州和韩国奉化郡等友好城市的联系，努力寻求合作机遇，提升合作层次。拓宽对外交往渠道，积极争取外援项目和资金。实施“走出去”和市场多元化战略，培育壮大外贸出口主体，鼓励企业与发达地区开展加工贸易合作，大力支持民营企业开展进出口贸易。引导具有资金、技术、管理优势的企业走出去，带动技术、设备、商品和劳务出口，提高经济发展的外向度。

　　鼓励引导全民创业。创业是发展之基、富民之本。我们要大力倡导全民创业、创新、创优，引导百姓创家业、能人创企业、干部创事业，激发全民的创业致富热情。建立健全创业孵化、创业培训、创业指导、创业扶持“四位一体’的机制，鼓励下岗失业人员、外出务工返乡人员、大中专毕业生、退役士兵创办企业。

　　（五）大力发展服务业，发挥消费对经济的拉动作用

　　我市服务业占生产总值的比重仅为37％，低于全国平均水平。差距就是潜力。我们一定要把加快发展服务业放在更加突出的位置，努力在发展传统服务业、培育现代服务业上实现新的突破，使服务业成为拉动全市经济增长的又一重要引擎。

　　加快发展旅游业。编制全市旅游业发展规划。推进药王山、玉华山风景名胜区开发，抓好照金红色旅游线路建设，加强耀州窑、陈炉陶瓷古镇的保护与开发，办好玉华冰雪节、药王养生节等活动，全年接待国内外游客346万人次，实现旅游综合收入4．2亿元。

　　提升服务业发展水平。以连锁经营和物流配送为重点，积极引进战略投资者，改造提升北市区商贸流通业，便利消费进社区，便民服务进家庭，满足群众多层次、多样化的消费需求。统筹南市区商业网点布局，制定加快新区服务业发展的决定，采取更加优惠的政策，大力扶持新区商贸流通、餐饮服务、文化娱乐、宾馆业发展，促使已形成的商业门店尽快开业，努力改善新区的商务环境。

　　努力扩大消费需求。全面落实鼓励消费的各项政策措施，提高居民的消费预期，扩大即期消费规模。加强农村市场体系建设，新建150家标准化农家店。大力支持连锁企业向农村延伸，推进绿色农产品进超市、进商店，促进农村消费提速。加快发展信息咨询、中介服务、旅游度假、美容健身等服务业。全年实现社会消费品零售总额29亿元，增长15%。

　　（六）狠抓增收节支，提高公共财政管理水平

　　坚持依法治税理财，优化财政收支结构，提高公共财政的保障能力。

　　促进财政增收。把开源的重点放在工业上，把挖潜的重点放在服务业上，大力支持成长性好、发展潜力大、带动能力强的企业发展，做强支柱财源，做大后续财源，积极培育新的税收增长点。依法治税，依率计征，做到应收尽收。规范行政事业性收费、价格调节基金、国有资本经营收益等非税收入管理，确保全面完成收入任务。

　　优化支出结构。按照保工资、保运转、保稳定、保重点的原则，从严控制一般性支出。全面深化部门预算改革，市级部门预算试点增加到28个。扩大国库集中支付范围，增强预算管理的透明度。加强政府债务管理，建立偿债准备金，从机制上防范和化解财政风险。强化预算执行审计、经济责任审计，规范财政资金使用行为。大力提倡增收节支，反对铺张浪费，提高资金使用效益。

　　增强金融支持力度。搭建银企合作平台，建立互信共赢的银企合作关系。抓紧国家开发银行陕西分行政府信用贷款项目的落实。加强社会信用体系建设，防范金融风险。鼓励银行开发新的金融产品，努力扩大信贷规模。

　　四、坚持以人为本，推进社会主义和谐社会建设

　　（一）强势推进创卫工作，努力改善人居环境

　　我市创卫工作已经到了决定胜负的关键时刻。要举全市之力，全民动员，人人动手，全面完成创卫任务，展示铜川的新形象！

　　全力打赢创卫攻坚战。市财政今年安排3500万元专项资金，用于创卫工作。按照“以块为主、条块结合，纵向到底、横向到边” 的要求，明确时限，责任到人，将创卫目标任务分解落实到每个部门和单位。广泛发动群众，大力开展宣传教育活动，努力提高市民素质，引导群众养成文明健康的生活习惯。调动社会各界参与创建卫生城市的积极性，广泛筹措资金，加快创卫硬件建设。建成新耀垃圾无害化处理一期工程，完成新耀污水归集管网建设任务，开工建设北市区生活垃圾无害化处理场，改扩建污水处理设施。新建、改造公厕107座，完成24个城中村和17个卫生村改造整治任务。深入开展市容环境综合整治，加大市场、背街小巷、城乡结合部市容管理力度，提高城市净化、绿化、亮化、美化水平。加强督促检查，坚持周检查、月考核工作机制，对连续两次考核排名靠后的单位予以通报批评、公开曝光，并实行责任追究。

　　改造提高北市区。要以闲置土地、公房等资产的置换开发为重点，强化经营城市的理念，聚合社会资金，加快城市建设步伐，改善人居环境。加快永乐堤南段、永祥堤北段道路景观综合改造，全面完成漆水河景观改造任务。改造金华路、频阳路和红旗桥、七一桥，建成正大、云梦、曙光等5个街头广场，更新人行道4万平方米，新增城市绿地12万平方米。开工建设集贤阁、川口粮站、北关桥头等标志性高层建筑，提升城市形象。

　　加快南市区开发。坚持“高水平是财富，低标准是包袱” 的发展理念，树立精品意识，加大新区开发建设力度，进一步完善新区建成区的功能。完成朝阳路、正阳路、长虹路非机动车道、人行道建设及绿化亮化工程，改造阳光广场，完善新区已建成道路信号灯、标志、标线等配套设施，新铺人行道10万平方米。实施长丰路、长虹北路、长丰市场南路照亮工程，完成南部工业园区排水、绿化亮化工程。市医院新区分院、新区体育馆投入使用，市一中新区校区、明德小学秋季开学招生。抓好新区的村庄改造试点，扶持新区农民发展服务业、建筑业等非农产业。美化绿化210国道耀州城区南大桥至泥阳段，完成永安北路改造任务，加大耀州城区旧城改造力度，完善沮河橡胶坝配套设施，加快沮河风景线和新耀连接带开发步伐，推进新耀一体化。

　　各位代表，创建省级卫生城市是我们自加压力，努力改善全市人居和发展环境的一项重大举措。铜川是我家，创卫靠大家。只要我们心往一处想，劲往一处使，我们就一定能够如期实现创建省级卫生城市的宏伟目标！

　　（二）加大资源和环境保护力度，增强可持续发展能力

　　我们一定要本着对未来发展、对子孙后代高度负责的精神，节约优先，环保优先，在全社会大力倡导节约、环保、文明的生产方式和消费模式，建设资源节约型、环境友好型社会，努力使铜川的天更蓝、地更绿、水更清。

　　狠抓节能降耗。坚持开发与节约并举，节能、节水、节地、节材，推进资源的综合利用。围绕循环经济上项目，围绕“三废”利用搞开发。启动声威、秦岭窑尾余热利用工程，推动水泥、铝冶炼、电力等行业的清洁生产。鼓励企业采用新技术、新工艺、新材料进行节能改造，坚决淘汰小水泥、小冶炼、小电石等落后产能。继续坚持能耗公报制度，严格落实目标考核和责任追究制度，加大对水泥、铝冶炼等重点耗能产业的监管力度。

　　加强环境污染治理。落实水泥企业污染治理方案，加快水泥粉尘污染治理步伐，再关闭拆除3条机立窑生产线，确保全市水泥企业达标排放。深化煤烟污染治理，完成北市区24台燃煤锅炉改造任务，将黄堡镇、耀州城区纳入煤烟污染治理范围。抓好沮河、漆水河水污染防治，加强水源地保护，保障群众饮水安全。严禁污染企业和城市污染物向农村扩散，控制农村面源污染。力争市区空气质量二级和好于二级的天数达到256天。

积极推进生态建设。按照“拆、治、刷、栽、控、整、管、教”八个方面的要求，综合整治210国道川口至耀州区沿线环境，全面完成沿线废弃厂房、危旧建筑物、构筑物和违章建筑的拆除任务。对川口至市水泥厂废弃矿山三个直观山崖断面实施立体绿化，210国道直观山坡重点区域造林2000亩，川口地区新栽乔木15万株。认真抓好“三化一片林”绿色家园、天然林保护、退耕还林、“三北”防护林四期等林业重点工程建设，全年植树造林15万亩，治理水土流失100平方公里。

合理开发利用资源。继续抓好矿产资源开发秩序的整顿，做好水泥灰岩、陶瓷粘土、油母页岩等资源的地质勘探工作，争取开展矿产资源有偿使用试点，促进煤层气、煤矸石、粉煤灰的综合开发利用。坚决实行最严格的土地管理制度，加强耕地特别是基本农田保护，守住全市耕地不少于124万亩这条红线。坚持集约节约用地，严格按照招拍挂方式提供工业、商业和房地产用地，严禁违法用地。盘活国有企业土地，动态储备土地200亩，保证重点项目用地需求。搞好土地开发整理，新增耕地3000亩，实现占补平衡。争取玉皇阁水库顺利交接，积极开展骨干水源、域外调水和水资源优化配置方案的论证，为经济社会发展提供可靠的水资源保障。

（三）大力发展社会事业，促进社会全面进步

把发展社会事业摆在构建和谐社会的重要位置，加大对社会事业的投入，着力提高社会事业的发展水平。

办好让人民满意的教育。坚持把教育放在优先发展的战略地位，促进各类教育均衡发展。全市财政安排教育支出2．76亿元，增长58％。全面启动“双高普九”，强化素质教育，确保全市教育“两基”工作顺利通过国家检查验收。落实好农村义务教育经费保障的各项政策，创建1个省级、2个市级教育强乡镇。完成55所中小学5．7万平方米危房改造任务，全面消除中小学D级危房。发挥铜川职业技术学院的龙头作用，整合职业教育资源，扩大职业教育和成人教育规模。实施“人人技能工程”，逐步使全市初、高中毕业未升学的学生，都能够进入职业学校接受培训。

做好医疗卫生和人口计生工作。全市财政安排医疗卫生支出6146万元，增长71.7％。继续抓好宜君县、耀州区、新区新型农村合作医疗试点，启动印台区、王益区的试点工作，全市农民参合率达到90％以上，建立市内报销直通车制度，在全市全面普及新型农村合作医疗制度。大力发展城市社区卫生，建立大医院与社区卫生服务机构双向转诊机制，逐步形成“小病在社区、大病进医院、康复回社区”的分级医疗服务格局，城市社区卫生服务覆盖面达到80％。完成市中医医院病房楼和宜君县人民医院、印台区妇幼保健站、耀州区孙思邈中医院以及16个乡镇卫生院建设项目，建成100个标准化村卫生室。利用卫生部健康快车为1000名白内障患者免费做复明手术。加强疾病预防控制和医疗救治体系建设，提高应对突发公共卫生事件的能力。稳定低生育水平，提高出生人口素质，建成1个县级、2个乡级标准化计生服务站。

繁荣文化体育事业。全市财政安排文化体育传媒支出3093万元，增长93．4％。加强公共文化设施建设，盘活川口影剧院闲置资产，开工建设新区图书馆，建成3个乡镇文化站、10个村级文化室。继续实施20户以上自然村“村村通”工程，新增数字电视用户2000户。开展进科普、送戏剧、送电影进农村、入社区活动，丰富城乡群众的文化生活。完成耀州文庙、药王山古建维修工程，力争开工建设耀州窑金元遗址保护大厅。加强农村和城市社区体育设施建设，广泛开展全民健身活动，办好第四届农民运动会。

实施人才强市战略。加大人才培养引进力度，抓好党政人才、经营管理人才和专业技术人才队伍建设。推进人事制度改革，完善人才评价、激励机制。继续实施农村基层人才队伍振兴计划，引导和鼓励高校毕业生到基层、到农村工作。采取按需选派、对口支援、定期轮换的方式，选派中级以上职称的专业技术人员进行为期一年的支农、支医、支教服务，为经济社会发展提供有力的人才保障。

（四）更加关注民主，促进社会和谐

社会和谐稳定，百姓安居乐业，是广大人民的共同愿望，更是政府工作的重要任务。今年市委、市政府继续为全市人民办20件实事，各区县、各部门也要为群众办一批暖民心、得民心的实事，让广大人民群众不断从和谐社会建设中得到实惠，共同建设、共同享有和谐社会！

千方百计扩大就业。全市财政安排社保就业支出1．31亿元，增长34．7％。认真落实新一轮促进就业再就业的各项扶持政策，完善扩大就业的长效机制，全年新增城镇就业1.26万人。扶持发展就业容量大的劳动密集型产业、民营经济、中小企业，鼓励和支持劳动者自主创业和自谋职业。深入开展创业促就业和就业援助活动，开发200个公益岗位，解决40岁以上的下岗失业妇女的就业问题，帮扶2000名就业困难人员实现再就业，确保剩余的652户零就业家庭至少有一人就业。建立企业工资保证金制度，解决企业拖欠职工工资，特别是拖欠克扣农民工工资问题。实施劳务富民工程，完善劳务输出服务体系，全年劳务输出4．5万人。

不断完善社会保障体系。继续扩大养老、医疗、失业、工伤和生育保险的覆盖面。完善社会统筹与个人账户相结合的基本养老保险制度，逐步做实个人账户。认真做好养老金计发新办法实施工作，稳步提高企业退休人员养老金发放标准。完善城镇职工基本医疗保险制度，努力解决企业未参加医疗保险的退休人员的参保问题。加大社保资金监管力度，提高资金使用的安全性和有效性。强化住房公积金归集，扩大公积金贷款，管好用好公积金，全年归集住房公积金1．2亿元，发放贷款4000万元。

着力改善困难群众生活。高度关注弱势群体，加大以扶老、助残、救孤、济困为重点的社会救助力度，让公共财政的阳光温暖更多的困难群众。全面落实农村低保制度，确保年人均纯收入低于625元的贫困户保障面超过70％，保障对象每月补差不低于25元。组建市老年大学，9O岁和百岁以上高龄老人生活保健补贴标准分别提高到每人每月100元和200元。落实农村五保供养政策，做到应保尽保、按标施保，提高五保户集中供养率。为残疾人办好十件实事，进一步改善残疾人的生存生活状况。完善城乡医疗救助、救灾救济体系，健全流浪乞讨人员救助机制，支持社会慈善事业发展。每年安排50万元专项救助资金，资助新录取的贫困家庭大学生入学。为3000户困难居民发放取暖补贴。加快五一路、小河沟、川口等棚户区改造步伐，拆除危旧房8万平方米，搬迁居民1000户；全市新开工住房26万平方米，建成20万平方米，其中廉价廉租房5万平方米，力争2000户住房困难群众入住新居。

（五）加强社会管理，构筑社会和谐基础

深入开展“三排查、三落实”工作，强化基层基础建设，切实改进社会管理，狠抓安全生产，维护社会和谐稳定。

大力推进社区建设。实施星光计划，安排专项资金，抓好全市57个社区“一部三室三站一场”等基础设施建设，把街道办事处、乡镇政府的社会管理和公共服务职能延伸到社区。提高社区干部的补贴标准，发挥社区在就业促进、帮困救助、人口管理、纠纷调解、安全保障工作中的作用，创建一批管理有效、服务完善、文明祥和的示范社区。

加强精神文明和民主法制建设。广泛开展社会主义荣辱观教育，提高市民道德素养，形成良好的社会风尚。深入贯彻监督法，自觉接受市人大及其常委会的法律监督和市政协的民主监督，认真办理人大代表、政协委员的建议和提案。继续抓好“五五”普法，搞好法律服务和法律援助。做好民兵预备役和国防动员工作，深入开展双拥共建活动，巩固双拥模范城创建成果。

高度重视安全生产。铜川是煤炭城市，又是连接南北、沟通东西的交通枢纽，安全工作责任重大。认真贯彻安全第一、预防为主、综合治理的方针，夯实安全生产责任，坚持不懈地开展煤矿及非煤矿山、道路交通、公共聚集场所、危险化学品、民爆物品和烟花爆竹等专项整治活动，打赢治理煤矿瓦斯和关闭3万吨以下小煤矿两个硬仗，严禁客运车超员和长途客车夜间行驶，严禁农用车违法载客，坚决遏制重特大事故的发生。强化食品、药品、餐饮质量安全监管，确保人民群众饮食用药安全。抓好防汛防滑工作，确保安全度汛。

切实维护社会稳定。落实信访工作责任，畅通诉求渠道，开展矛盾纠纷排查化解活动，坚决纠正征地拆迁、企业改制、环境保护中损害群众利益的行为，实现信访工作“一控制、两下降”目标。加强应急管理工作，建立健全分类管理、分级负责、条块结合、属地为主的应急管理体系，提高保障公共安全和处置突发事件的能力。深入开展平安铜川创建活动，加强基层政法“两所一庭”建设，完善社会治安防控体系，严厉打击违法犯罪行为，增强人民群众的安全感。

各位代表，安全为天，稳定压倒一切。我们要坚持做到第一要务抓发展，第一责任保稳定，把安全生产和维护稳定的各项政策措施切实落到实处，努力使全市人民生活得更加幸福、安康！

五、大力推进政府自身建设

认真学习贯彻胡锦涛总书记给延安人民的复信精神，为全市经济社会又好又快发展提供强大的精神动力。要进一步增强忧患意识、公仆意识、节俭意识，按照作风建设年的总体部署，以提高行政效能为核心，大力推进政府管理创新和作风建设，努力建设行为规范、公正透明、勤政高效、清正廉洁的政府，建设一个人民群众满意的政府！

　　（一）转变政府职能。从管理理念、管理体制和管理方式上改革政务，改进政府管理和服务方式，发挥市场在资源配置中的基础性作用。强化社会管理和公共服务职能，增强基本公共服务能力。深化行政管理体制改革，减少和规范行政审批事项。调整和优化政府组织结构与职责分工，加强编制的动态管理，对职能变化、业务量减少、效能低下的部门和单位缩减编制，优化人力资源配置。创新经费管理模式，实行以奖代补，经费与业绩挂钩，财政投入向能干难事、善干新事、敢干大事的部门和单位倾斜。贯彻落实行政许可法，加大政务公开力度，加快推进电子政务和政府同站建设，完善政府新闻发布制度，拓宽社情民意反映渠道，增强政府工作的透明度和人民群众的参与度，真正做到让群众知情、让群众参与、让群众满意。

（二）推进依法行政。严格依照法定权限和程序行使权力、履行职责、接受监督，切实将政府管理经济社会的行为纳入法制化轨道。健全行政决策机制，完善重大事项集体决策、专家咨询、社会公示与听证等制度，推进决策的民主化、科学化。严格执行行政执法责任制、行政问责制、行政执法过错责任追究制，坚决治理行政不作为、乱作为的行为，真正做到有权必有责、用权受监督、侵权须赔偿、违法受追究。

（三）改进工作作风。积极倡导“八个方面”的良好风气，弘扬求真务实精神，大兴求真务实之风，讲实话，办实事，求实效，真正做到权为民所用，情为民所系，利为民所谋。树立实干就是能力、落实就是水平的理念，健全抓落实的工作机制，加大督促检查和考核力度，确保工作有部署、有检查、有奖惩，全面提高行政效能，增强政府的执行力和公信力。大力精简会议，压缩文件，改进会风、文风，严格控制和规范各种检查、评比、达标活动。加强公务员队伍建设，健全公务员政绩考核评价体系，强化目标绩效管理，奖优、治庸、罚劣。完善公务员培训制度，通过在职培训、挂职锻炼、岗位轮换等方式，不断提高公务员综合素质。

（四）加强廉政建设。认真落实廉洁自律的各项规定，坚持标本兼治、综合治理、惩防并举、注重预防的方针，完善教育、制度、监督并重的惩治和预防腐败体系。加强监察和审计工作，畅通监督渠道，强化对权力运行的制约和监督。深入开展治理商业贿赂活动，抓好领导干部经济责任审计，加强对政府采购、经营性土地招拍挂、产权交易、建设工程招投标等重点领域和环节的监管，从源头上预防和治理腐败。时刻牢记“两个务必”，大力发扬艰苦奋斗、勤俭节约的精神，坚决反对铺张浪费、大手大脚等奢侈之风。规范公务消费行为，堵塞漏洞，努力降低行政成本，建设节约型政府。政府工作人员要严于律己，公道正派，当好公仆，真正做到“为民、务实、清廉”。

各位代表，建设以现代建材业为主导的新型工业城市，实现铜川率先发展跨越发展的宏伟目标，是时代赋予我们的光荣使命。让我们更加紧密地团结在以胡锦涛同志为总书记的党中央周围，在市委的坚强领导下，锐意进取，励精图治，努力谱写铜川改革发展的新篇章！
